# Does the consumption of fruits and vegetables differ between Eastern and Western European populations? Systematic review of cross-national studies

**DOI:** 10.1186/s13690-015-0078-8

**Published:** 2015-06-15

**Authors:** Denes Stefler, Martin Bobak

**Affiliations:** Department of Epidemiology and Public Health, University College London, 1-19 Torrington Place, London, WC1E 6BT UK

**Keywords:** Fruit and vegetable consumption, Central and Eastern Europe, Former Soviet Union, Cross-national studies

## Abstract

**Background:**

Difference in fruit and vegetable consumption has been suggested as a possible reason for the large gap in cardiovascular disease (CVD) mortality rates between Eastern and Western European populations. However, individual-level dietary data which allow direct comparison across the two regions are rare. In this systematic review we aimed to answer the question whether cross-national studies with comparable individual-level dietary data reveal any systematic differences in fruit and vegetable consumption between populations in Central and Eastern Europe (CEE) and the Former Soviet Union (FSU) compared to Western Europe (WE).

**Methods:**

Studies were identified by electronic search of MEDLINE, EMBASE and Web of Science databases from inception to September 2014, and hand search. Studies which reported data on fruit, vegetable consumption or carotene and vitamin C intake or tissue concentrations of adult participants from both CEE/FSU and WE countries were considered for inclusion. Quality of the included studies was assessed by a modified STROBE statement. Power calculation was performed to determine the statistical significance of the comparison results.

**Results:**

Twenty-two studies fulfilled the inclusion criteria. Fruit consumption was found to be consistently lower in CEE/FSU participants compared to Western Europeans. Results on vegetable intake were less unambiguous. Antioxidant studies indicated lower concentration of beta-carotene in CEE/FSU subjects, but the results for vitamin C were not consistent.

**Conclusion:**

This systematic review suggests that populations in CEE and FSU consume less fruit than Western Europeans. The difference in the consumption of fruit may contribute to the CVD gap between the two regions.

**Electronic supplementary material:**

The online version of this article (doi:10.1186/s13690-015-0078-8) contains supplementary material, which is available to authorized users.

## Background

Cardiovascular disease (CVD) mortality rates are considerably higher in countries of Central and Eastern Europe (CEE) and Former Soviet Union (FSU) compared Western Europe (WE) [1]. Differences in diet quality between the two regions, fruit and vegetable consumption in particular, has been one of the proposed explanations for this health gap [2–5].

The lack of internationally comparable, individual-level dietary data in Europe is a well-known problem in public health nutrition [6–9]. In 2011, the European Food Safety Authority (EFSA) published the Comprehensive European Food Consumption Database of food consumption data for most EU member states collected by national dietary surveys of individual-level intakes. However, the authors emphasised that due to the differences in data collection methods, the database was not suitable for international comparisons [10]. Other than the differences in dietary assessment methods, the lack of uniform food-grouping and coding system, and differences in estimated portion sizes and food composition tables also make the nationally collected and analysed dietary data inadequate for direct country-to-country comparison [7, 8, 11].

Previous systematic reviews of fruit, vegetable and micronutrient intakes in CEE, FSU and WE countries used data from studies which had been conducted separately in the two regions [12, 13]. These reviews found that the methodological differences between studies seriously limited the interpretation of the results, and emphasised that the lack of comparable data was especially important in CEE and FSU countries. In this respect, cross-national studies which include participants from both CEE/FSU and WE countries, and collect and analyse dietary data in a standardized way, may be therefore more suitable for direct comparisons of food intakes between the two regions.

The aim of this work was to systematically review cross-national studies which reported individual-level data on consumption of fruits, vegetables, or their indicators, such as vitamin C and carotenoids, of participants from CEE/FSU and WE populations using identical methods for data collection and analysis in the two regions.

## Methods

### Search strategy

MEDLINE, EMBASE and Web of Science databases were searched from inception to September 2014, using search terms described in Appendix 1. References and citation lists of selected papers were studied for additional papers, and hand search of key journals (*Public Health Nutrition, European Journal of Clinical Nutrition, European Journal of Public Health*) was also performed. No restriction on language was applied.

### Inclusion and exclusion criteria

Original, quantitative, observational epidemiological studies which described fruit, vegetable, antioxidant intakes or antioxidant status of adult participants who live in CEE or FSU countries and provided comparison populations from Western Europe were included in the review. Based on the data collection methods and reported dietary data, the following studies were considered for inclusion: (1) Dietary surveys: studies which reported data on fruit and vegetable intake levels using established nutritional assessment methods such as food frequency questionnaire (FFQ), diet history, dietary record and 24-h diet recall. (2) Health behavioural surveys: reporting data on fruit and vegetable intakes using lifestyle questionnaires with questions regarding fruit or vegetable consumption habits. (3) Antioxidant studies: reporting data on average vitamin C or carotenoid intakes or status (including plasma, serum and adipose tissue concentrations).

Studies were excluded if data collection methods or the inclusion criteria of participants differed substantially between the two regions. Studies which compared dietary habits between the former East and West Germany were used only if their data collection took place before 1991, because food consumption patterns of East Germans seem to have changed rapidly after the reunification [14].

To avoid bias towards studies which reported more than one exposure of interest from the same participants, we included only one set of data from these studies in the review: data on carotenoid and vitamin C intake or status were included only if no data on fruit or vegetable consumption were available. If both antioxidant intake and status were reported, only intake data was used, and if data on more than one type of carotenoid concentration were available, only beta-carotene was extracted.

### Quality assessment

Quality of the included studies was assessed by a shortened version of the Strengthening the Reporting of Observational Studies in Epidemiology (STROBE) statement [15]. Modification of the checklist was necessary because several studies described only the nutritional characteristics of the subjects and the analysis of the relationship with disease outcomes was not reported. Therefore four items of the statement, which refer to the variables and outcome results of an analytic study (item nos. 7, 11, 15 and 16), were omitted and the assessment was carried out using the remaining 18 items.

### Data analysis

Most studies described dietary data of participants from more than one country within a certain region. For these studies, the average values for CEE/FSU and WE were calculated and reported in the review.

To take into account the well-documented difference in fruit and vegetable consumption between Northern and Southern European countries [16, 17], both CEE/FSU and WE regions were divided into “south” and “north” sub-regions (Table 1). If a study reported g/day intake levels of fruits or vegetables of participants from opposite sub-regions, north/south weighting was applied: the intake figure of the “south” country was multiplied with a weighting factor calculated from FAO data [18] by dividing the average fruit or vegetable supply of all northern countries of that region between 1970 and 2009 by the specific country’s average supply over the same time period. For studies reporting data on the percentages of participants eating daily fruits or vegetables, or antioxidant data, no such weighting was carried out because appropriate weighting factors were not available.

**Table 1:** Grouping of Central and Eastern European (CEE)/former Soviet Union (FSU) and Western European (WE) countries

Region	Sub**-**region	Countries
CEE/FSU	North	Armenia, Azerbaijan, Belarus, Czech Republic, Estonia, Georgia, Hungary, Kazakhstan, Kyrgyzstan, Latvia, Lithuania, Poland, Republic of Moldova, Romania, Russian Federation, Slovakia, Tajikistan, Turkmenistan, Ukraine, Uzbekistan
	South	Albania, Bosnia and Herzegovina, Bulgaria, Croatia, Montenegro, Serbia, Slovenia, TFYR Macedonia
WE	North	Austria, Belgium, Denmark, Finland, France, Germany, Iceland, Ireland, Liechtenstein, Luxembourg, Netherlands, Norway, Sweden, Switzerland, United Kingdom
	South	Andorra, Greece, Italy, Portugal, San Marino, Spain

If data were collected in winter or spring months in one region and during summer or autumn in the other, seasonal weighting of the CEE/FSU data was applied: the intake figures were multiplied with a weighting factor which was calculated from the Health Alcohol and Psychosocial Factors in Eastern Europe (HAPIEE) study, which is the largest study in CEE/FSU with dietary data [19]. The weighting factor was determined as the ratio of the energy standardized mean intake level between participants who completed the questionnaire in the summer/autumn months and those who completed it during the winter or spring months. Weighting for seasonal variation was applied only in CEE/FSU because seasonal differences in this region are more substantial than in Western Europe [5, 20, 21].

Most reviewed studies did not report statistical significance of the differences between CEE/FSU and WE. In order to assess whether the reported differences were statistically significant, power calculation was applied. If a study had more than 80 % power to show the described difference as statistically significant on the 0.05 significance level, we considered the reported difference statistically significant. If the power was between 20 % and 80 %, we considered that the observed difference was non-significant but the trend was worth noting, and if the power was lower than 20 %, the difference was considered negligible. Power calculations were carried out using STATA 12.1 statistical software (StataCorp Texas, USA).

If standard deviation (SD) value was required for power calculation but it was not available from the specific study [22–27], the average SD of fruit, vegetable, vitamin C and beta-carotene intake and concentration levels reported in the European Prospective Investigation into Cancer and Nutrition (EPIC) study cohorts was assumed [16, 28]. We considered this assumption appropriate because EPIC is the largest international study with such data available and its results suggest that SD values vary in a narrow range irrespectively of study size and mean intake level. In the study which measured adipose tissue beta-carotene concentration [29] the SD reported on a subsample of the same study participants were used [30]. In studies where south/north or seasonal weighting was applied, SD values were multiplied with the same figures as the mean values.

## Results

### Characteristics of the reviewed studies

**Fig. 1:**
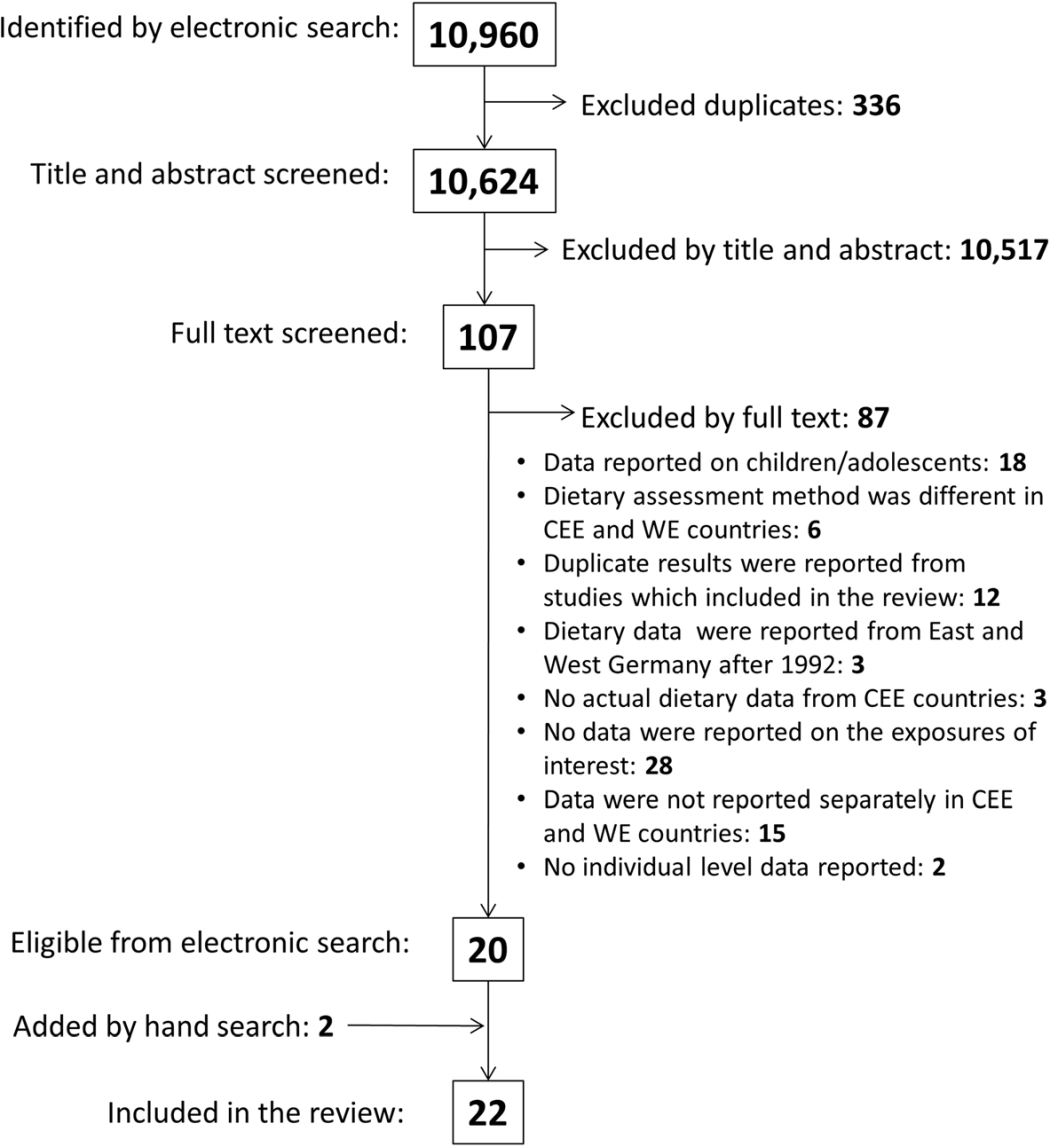
Flow diagram of the study selection process

Twenty-two studies met the inclusion criteria: ten dietary surveys [22–26, 31–35], six health behavioural surveys [36–41] and six antioxidant studies [27, 29, 42–45]. Fig. 1 shows the study selection process and Table 2 (see Additional file 1) describes the main features of the included studies. Most studies were cross-sectional in design or reported cross-sectional data from cohort studies. In two studies [29, 32], data were extracted from case–control setting. Participants from 18 CEE/FSU countries and 18 WE states were included in the comparisons and most countries were covered by more than one study. The earliest study [22] reported data from the early 1960s, while the latest data collection took place in 2010 [41]. Sample sizes ranged from 30 to 85 921 per region. Five studies [22, 29, 31, 42, 43] recruited only males but the majority gave dietary data for both genders. More than half of the studies applied random sampling method at recruitment and eight [26, 33, 37–40, 43, 45] used the general population as the sampling frame.

**Table 2:** Characteristics of included studies

1^st^ author, year of publication	Name of study	Examined food or antioxidant	Dietary assessment	Participants’ country of origin		Year of data collection	Month of data collection	Sample size	Response rate (%)	Females (%)	Age range or mean (years)	Sampling method	Basis of sample	Quality score* (max:18)
**1. DIETARY SURVEYS**														
Kromhout 1989 [22]	Seven Countries Study	Fruits, vegetables	7d record	CEE:	Yugoslavia	1960-64	Jan-May, Sep	150	nd	0	40-59	random	farm/factory workers, academics	9
				WE:	Finland, Italy, Greece Netherlands	1959-65	Feb-Sep	286	nd	0	40-59	random	village inhabitants, railroad workers	
Winkler 1992(31]		Fruits, vegetables	3d record	CEE:	GDR	1987	Oct-Dec	132	73	0	45-64	random	urban inhabitants	11
				WE:	FDR	1984-85	Oct-May	424	70	0	45-64	cluster	urban inhabitants	
Schroll 1996 [23]	SENECA	Fruits, vegetables	Diet history	CEE:	Poland	1993	Jan-Jun	120	51†	61	74-79	random	urban inhabitants	13
				WE:	Belgium, Denmark, France, Italy, Netherlands, Portugal, Spain, UK, Switzerland	1993	Jan-Jun	1237	51†	51	74-79	random	urban inhabitants	
Karamanos 2002 [24]		Fruits, vegetables	Diet history	CEE:	Bulgaria	nd	nd	288	nd	50	35-60	random	urban inhabitants	14
				WE:	Italy, Greece	nd	nd	1058	nd	54	35-60	random	urban and rural inhabitants	
Serra-Majem 2003 [25]	WHO-CINDI	Fruits, vegetables	24hr recall	CEE:	Poland	1991-94	nd	4440	nd	50	20-65	random	factory workers	14
				WE:	Spain	1992	nd	2757	69	nd	6-75	random	general population	
Petkeviciene 2009 [26]	NORBAGREEN	Fruits, vegetables	FFQ	CEE:	Lithuania	2002	Apr	99	68	57	19-75	random	general population	15
				WE:	Finland	2002	Jan-May	125	91	nd	25-64	random	general population	
Lixandru 2010 [32]		Fruits, vegetables	FFQ	CEE:	Romania	2005	Apr-Nov	40	nd	30	63	convenience	diabetic patients	12
				WE:	Belgium	2005	Apr-Nov	30	nd	20	62	convenience	diabetic patients	
Paalanen 2011 [33]		Fruits, vegetables	FFQ	CEE:	Russia	1992-07	Mar-May	2672	45-92	57	25-64	random	general population	16
				WE:	Finland	1992-02	Mar-May	4365	67-81	53	25-64	random	general population	
Crispim 2011 [34]	EFCOVAL	Fruits, vegetables	24hr recall	CEE:	Czech Republic	2007-08	Oct-Apr	118	nd	51	45-65	convenience	healthy individuals	16
				WE:	Belgium, France, Norway Netherlands,	2007-08	Apr-Jul, Oct-Apr	482	nd	50	45-65	convenience	healthy individuals	
El Ansari 2012 [35]	CNSHS	Fruits, vegetables	FFQ	CEE:	Bulgaria, Poland	2005	nd	1143	95	70	21	convenience	university students	14
				WE:	Denmark, Germany	2005	nd	1236	85-92	53	21	convenience	university students	
**2. HEALTH BEHAVIOUR SURVEYS**														
Wardle 1997 [36]	EHBS	Fruits	na	CEE:	Poland, Hungary, GDR	1989-92	nd	2293	90-100	51	22	convenience	university students	13
				WE:	Austria, Belgium, FDR, UK Denmark, Finland, Spain, France, Greece, Iceland, Ireland, Italy, Sweden, Netherlands, Norway, Portugal, Switzerland	1989-92	nd	14192	90-100	56	21	convenience	university students	
Prattala 2007 [37]	Finbalt Health Monitor project	Fruits	na	CEE:	Estonia, Latvia, Lithuania	1998-02	Apr-May	15740	62-80	57	20-64	random	general population	16
				WE:	Finland	1998-02	Apr-May	9354	65-70	53	20-64	random	general population	
Prattala 2009 [38]	EUROTHIENE	Vegetables	na	CEE:	Estonia, Latvia, Lithuania	2000-04	nd	14219	60-73	58	20-64	random	general population	15
				WE:	Finland, Denmark, Spain, Germany, France, Italy	1998-04	nd	86924	61-87	51	20-64	random	general population	
Hall 2009 [39]	WHS	Fruits, vegetables	na	CEE:	Bosnia and Herzegovina, Croatia, Czech Republic, Estonia, Georgia, Hungary, Kazakhstan, Latvia, Russia, Slovakia, Slovenia, Ukraine	2002-03	nd	22475	69-100	53	18-99	random	general population	15
				WE:	Spain	2002-03	nd	5448	86	60	18-99	random	general population	
European Commission 2013 [40]	EHIS	Fruits, vegetables	na	CEE:	Bulgaria, Czech Republic, Estonia, Latvia, Hungary, Poland, Romania, Slovakia, Slovenia	2006-09	nd	85921	56-89	53	15-99	random	general population	na
				WE:	Belgium, Greece, Spain, France	2006-09	nd	62700	60-96	55	15-99	random	general population	
Burisch 2014 [41]	ECCO-EpiCom	Fruits, Vegetables	na	CEE:	Croatia, Czech Rep, Estonia, Hungary, Lithuania, Moldova, Romania, Russia	2010	Jan-Dec	249	76†	42	15+	convenience	IBD patients (at diagnosis)	16
				WE:	Cyprus, Denmark, Finland, Greece, Iceland, Ireland, Israel, Italy, Portugal, Spain, Sweden, UK	2010	Jan-Dec	933	76†	46	15+	convenience	IBD patients (at diagnosis)	
**3. ANTIOXIDANT STUDIES**														
Kardinaal 1993 [29]	EURAMIC	Beta-carotene in adipose tissue	na	CEE:	Russia	1991-92	nd	200	79-97	0	51	convenience	hospital patients, healthy controls	16
				WE:	Finland, Germany, Netherlands, Norway, UK, Spain, Switzerland	1991-92	nd	1180	50-98	0	54	convenience	hospital patients, healthy controls	
Kristenson 1997 [42]	LiVicordia	Beta-carotene in plasma	na	CEE:	Lithuania	1993-94	Oct-Jun	100	83	0	50	random	urban inhabitants	14
				WE:	Sweden	1993-94	Oct-Jun	95	83	0	50	random	urban inhabitants	
Bobak 1998 [27]		Beta-carotene in plasma	na	CEE:	Czech Republic	1992	Sep-Nov	136	70	49	40-59	random	urban inhabitants	14
				WE:	UK	1991-93	nd	358	73	31	40-59	random	civil servants	
Bobak 1999 [43]		Beta-carotene in plasma	na	CEE:	Czech Republic	1995	Apr-Jun	188	70	0	45-64	random	general population	17
				WE:	Germany	1995	Apr-Jun	153	70	0	45-64	random	general population	
Miere 2007 [44]		Vitamin C intake	24h recall	CEE:	Romania	nd	nd	312	nd	87	21	convenience	university students	8
				WE:	Spain	nd	nd	918	nd	58	22	convenience	university students	
Woodside 2013 [45]	EUREYE	Vitamin C and Beta-carotene in plasma	na	CEE:	Estonia	2000-03	nd	833	58.6	66	65+	random	general population	15
				WE:	Norway, UK, France, Italy, Greece, Spain	2000-03	nd	3300	36-56	52	65+	random	general population	

WHO-CINDI, World Health Organization Countrywide Integrated Non-communicable Disease Intervention; NORBAGREE, Consumption of vegetables and fruits and other dietary health indicator foods in the Nordic and Baltic countries; EFCOVAL, European Food Consumption Validation; CNSHS, Cross National Student Health Survey; EHBS, European Health and Behaviour Survey; WHS, World Health Survey; EHIS, European Health Interview Survey; EURAMIC, European Community Multicentre Study on Antioxidants, Myocardial Infarction and Breast Cancer; LiVicordia, Linkoping-Vilnius Coronary Disease Risk Assessment Study; ECCO-EpiCom, European Crohn’s and Colitis Organization’s Epidemiological Committee study; FDR, Federal Republic of Germany; GDR, German Democratic Republic; CEE: Central and Eastern Europe (or Former Soviet Union); WE, Western Europe; FFQ, Food frequency questionnaire; na, not applicable; nd, no data available; IBD, Inflammatory bowel disease

*Based on evaluation using a modified STROBE checklist; †Overall response rate

Overall, the quality of the reviewed studies was good. 15 studies scored 14 or more points on the 18 point scale and only two [22, 44] scored less than ten points. Quality of one study [40] was not assessed because it was published as an online database, with no peer-reviewed research paper available.

### Findings of the reviewed studies

Table 3 (see Additional file 2) shows the average intake, percentage and concentration values of CEE/FSU and WE participants regarding fruit, vegetable and antioxidants reported by the reviewed studies. The directions of the observed differences and the extent of their significance, determined by power calculation, are also summarised.

**Table 3:** Summary results of the included studies

1^st^ author, year of publication	Unit of measurement	Sex	CEE countries			WE countries			Power	Summary: CEE compared to WE‡
			Average intake, cc. or %	Range*	SD	Average intake, cc. or %	Range*	SD		
**1. DIETARY SURVEYS**										
**FRUITS**										
Kromhout 1989 [22]§│	g/day intake	M	58.6	1.0-153.6	207.3†	132.1	21.3-310.9	178.3†	0.96	**LOWER**
Winkler 1992 [31]	g/day intake	M	98.0		145.3	101.0		164.3	0.05	no difference
Schroll 1996 [23]§	g/day intake	M	186.0		239.1†	234.0	120.0-532.5	230.2†	0.26	lower-ns
		F	162.0		210.2†	208.0	135.0-399.6	202.4†	0.43	lower-ns
Karamanos 2002 [24]	g/day intake	M	293.0		239.1†	315.0	236.0-355.0	239.1†	0.16	no difference
		F	303.0		210.2†	325.7	234.0-377.0	210.2†	0.21	lower-ns
Serra-Majem 2003 [25]§	g/day intake	M+F	137.0		224.7†	290.0		218.0†	1.00	**LOWER**
Petkeviciene 2009 [26]	p/month intake	M+F	20.8		84.3†	29.4		84.3†	0.12	no difference
Lixandru 2010 [3]	% eat daily	M	100.0		na	89.5		na	0.34	higher-ns
		F	100.0		na	100.0		na	na	no difference
Paalanen 2011 [33]	% eat daily	M	14.0	2.0-31.0	na	52.3	43.0-61.0	na	1.00	**LOWER**
		F	26.0	4.0-50.0	na	73.3	66.0-82.0	na	1.00	**LOWER**
Crispim 2011 [34]	g/day intake	M	207.0		176.7	197.0	163.0-228.0	175.1	0.07	no difference
		F	226.0		155.7	230.5	194.0-265.0	151.1	0.05	no difference
El Ansari 2012 [35]	% eat daily	M	31.6	23.8-39.4	na	30.4	28.6-32.1	na	0.05	no difference
		F	46.8	39.5-54.1	na	51.6	47.8-55.4	na	0.42	lower-ns
**VEGETABLES**										
Kromhout 1989 [22]§│	g/day intake	M	240.0	159.0-276.0	198.2†	102.6	57.3-227	88.1†	1.00	**HIGHER**
Winkler 1992 [31]	g/day intake	M	126.0		154.8	124.0		154.8	0.05	no difference
Schroll 1996 [23]§	g/day intake	M	341.0		154.8†	288.0	82.4-461.0	128.1†	0.63	higher-ns
		F	297.0		143.9†	238.0	77.0-383.0	121.0†	0.92	**HIGHER**
Karamanos 2002 [24]	g/day intake	M	243.0		154.8†	189.0	168.0-214.0	154.8†	0.96	**HIGHER**
		F	291.0		143.9†	197.3	178.0-222.0	143.9†	1.00	**HIGHER**
Serra-Majem 2003 [25]§	g/day intake	M+F	288.0		149.4†	97.1		68.7†	1.00	**HIGHER**
Petkeviciene 2009 [26]	p/month intake	M+F	29.9		56.0†	29.1		56.0†	0.05	no difference
Lixandru 2010 [32]	g/day intake	M	287.0		189.4	269.9		108.1	0.07	no difference
		F	258.3		157.9	283.3		125.2	0.06	no difference
Paalanen 2011 [33]	% eat daily	M	15.0	10.0-24.0	na	48.7	44.0-54.0	na	1.00	**LOWER**
		F	22.3	11.0-35.0	na	70.7	69.0-72.0	na	1.00	**LOWER**
Crispim 2011 [34]	g/day intake	M	162.0		121.1	201.0	168.0-222.0	112.8	0.60	lower-ns
		F	157.0		99.1	202.3	166.0-254.0	108.5	0.87	**LOWER**
El Ansari 2012 [35]	% eat daily	M	37.8	23.9-51.6	na	24.4	23.3-25.4	na	0.99	**HIGHER**
		F	44.9	28.0-61.8	na	42.0	37.5-46.4	na	0.18	no difference
**2. HEALTH BEHAVIOUR SURVEYS**										
**FRUITS**										
Wardle 1997 [36]	% eat daily	M	40.0	36.0-45.0	na	42.9	23.0-78.0	na	0.43	lower-ns
		F	65.0	59.0-74.0	na	61.1	36.2-86.0	na	0.72	higher-ns
Prattala 2007 [37]	% eat daily	M	11.0	10.0-12.0	na	18.0		na	1.00	**LOWER**
		F	20.3	17.0-25.0	na	36.0		na	1.00	**LOWER**
EHIS 2013 [40]	% eat daily	M	52.8	39.4-66.8	na	60.6	57.9-66.0	na	1.00	**LOWER**
		F	67.0	49.2-82.3	na	69.1	62.3-74.5	na	1.00	**LOWER**
Burisch 2014[41]	% eat daily	M+F	43.4		na	54.3		na	0.87	**LOWER**
**VEGETABLES**										
Prattala 2009 [38]	% eat daily	M	22.5	16.1-27.5	na	32.1	24.7-39.1	na	1.00	**LOWER**
		F	30.4	25.0-33.4	na	45.9	36.9-59.1	na	1.00	**LOWER**
EHIS 2013 [40]	% eat daily	M	54.8	44.2-71.3	na	68.6	56.0-82.7	na	1.00	**LOWER**
		F	62.5	55.0-78.6	na	74.2	65.3-87.4	na	1.00	**LOWER**
Burisch 2014 [41]	% eat daily	M+F	49.0		na	60.1		na	0.88	**LOWER**
**FRUITS AND VEGETABLES**										
Hall 2009 [3]	% eat >=5 p/day	M	18.1	8.0-44.5	na	22.0		na	0.98	**LOWER**
		F	23.5	9.4-49.7	na	24.9		na	0.38	lower-ns
**3. ANTIOXIDANT STUDIES**										
**BETA CAROTENE**										
Kardinaal 1993 [29]	ug/g fatty acid	M	0.51	0.45-0.56	0.80	0.42	0.18-0.59	0.80	0.31	higher-ns
Kristenson 1997 [42]	umol/l cc.	M	0.38		0.20	0.51		0.32	0.92	**LOWER**
Bobak 1998 [27]	umol/l cc.	M	0.39		0.26†	0.77		0.26†	1.00	**LOWER**
		F	0.52		0.40†	0.97		0.40†	1.00	**LOWER**
Bobak 1999 [43]	umol/l cc.**	M	0.11		0.08	0.20		0.21	1.00	**LOWER**
Woodside 2013 [45]	umol/l cc	M	0.25		0.26	0.34	0.19-0.48	0.31	1.00	**LOWER**
		F	0.36		0.34	0.44	0.30-0.67	0.37	1.00	**LOWER**
**VITAMIN C**										
Miere 2007 [44]	mg/day intake	M	80.3		54.8	106.2		83.4	0.77	lower-ns
		F	88.8		67.9	124.4		94.8	1.00	**LOWER**
Woodside 2013 [45]	umol/l cc.	M	42.0		23.8	38.0	32.7-44.4	23.1	0.74	higher-ns
		F	54.5		27.7	48.5	43.5-52.4	23.4	1.00	**HIGHER**

M, Males; F, Females; p, portion; EHIS, European Health Interview Survey; na, not applicable; cc., concentration

*Range of intake levels, percentages or concentrations if data was reported from more than one country or site

†SD assumed from EPIC study

‡**LOWER**: Intake level, percentage or concentration significantly **lower** in CEE/FSU countries compared to data from WE, (power > 0.80); **HIGHER**: Intake level, percentage or concentration significantly **higher** in CEE/FSU countries compared to data from WE, (power > 0.80); **lower-ns**: Intake level, percentage or concentration **lower** in CEE/FSU but difference not significant (power < 0.80 and >0.20); **higher**-**ns**: Intake level, percentage or concentration **higher** in CEE/FSU but difference not significant (power < 0.80 and >0.20); **no difference**: power < 0.20

§:North–south weighting was applied

I:Seasonal weighting was applied

**Calculated from reported data using molar mass = 537 g

Most studies reported their results separately for fruits and vegetables and for males and females. Majority of dietary surveys gave average fruit or vegetable consumption values as mean gram per day intakes, and most of the health behavioural surveys as the percentage of the sample who eat these foods at least once a day.

Regarding fruit intake, both dietary and health behavioural surveys showed consistently lower intakes in CEE/FSU compared to WE. Although six out of nine dietary survey comparisons with adequate power found higher vegetable intake in CEE/FSU countries, the estimates were consistently lower in health behavioural surveys. All antioxidant studies indicated lower concentration of beta-carotene in CEE/FSU subjects, but the results for vitamin C were not consistent. No consistent difference was found between males and females.

## Discussion

This systematic review of cross-national studies on fruit and vegetable intake found consistently lower fruit intake figures in CEE/FSU populations compared to WE, but no consistent difference for vegetable intake between the two regions.

Our results are congruent with ecological dietary data of food availability based on food balance sheets (FBS) and household budgetary surveys (HBS). Comparison of average fruit and vegetable supply in CEE/FSU and WE countries between 1970 and 2009 suggests clear difference only for fruits but not for vegetables [18]. Similarly, comparison of HBS data from DAFNE database indicates that, on average, the availability of fruits is lower but vegetables is higher in CEE/FSU countries [46].

The inconsistency of our findings regarding vegetable intake can be due to the lack of north/south weighting of health behavioural survey results. For example, in the European Health Interview Survey (EHIS), the largest health behavioural survey included in the review, most participants came from southern countries of Western Europe and northern part of CEE/FSU. If, as a sensitivity analysis, we applied the weighting factors calculated from FAO database for the EHIS results, the comparison showed that the proportion of individuals who consumed vegetables at least once a day was higher in CEE/FSU countries, which is similar to most dietary surveys.

On the other hand, most health behaviour surveys had larger sample size than the dietary surveys, and they are also less prone to measurement error. Furthermore, since the main food sources of beta-carotene are vegetables [47], the findings of the antioxidant studies are also in support of the health behavioural survey results and the lower vegetable intake in Eastern Europe.

On the whole, we cannot exclude the possibility that the reason for the inconsistent results regarding vegetable consumption is that there is no actual difference in intake between CEE/FSU and WE populations.

Our review has several limitations. Firstly, it is possible that further published or non-published studies exist which we did not identify during the search. However, cross-national studies tend to require substantial funding, logistics and international cooperation between institutions, which often go hand in hand with the endeavour to publish the work in internationally reputable journals which can be found in the electronic databases we searched. In addition, as we applied no language restriction in the electronic search, the possibility of finding studies from non-English speaking countries was increased.

Secondly, our data analysis involved several assumptions. The weighting factors from FAO database and HAPIEE study were the best options currently available for these purposes, and the SD values brought over from EPIC study did not influence the direction of the results, it only helped to decide whether the studies were sufficiently large to draw meaningful conclusions of their findings.

Although the reviewed studies included participants from a large number of CEE/FSU and WE countries, some of them providing nationally representative food consumption data, specific comparisons were representative only for a small proportion of the whole CEE/FSU and WE populations. Because large differences exist in fruit and vegetable intakes within the regions, the reported comparisons can only be seen as pixels of a much larger picture. The complete picture will emerge only when nationally representative, comparable dietary data is available for most European countries; in fact, this is the main aim of EFSA’s on-going “EU Menu” project [48].

## Conclusion

This systematic review supports previous data that people in CEE/FSU countries consume less fruit than Western Europeans, and that the difference in vegetable intake is probably less clear-cut. Since inadequate consumption of fruit is suggested as a modifiable risk factor for CVD [49, 50], the difference in fruit intake may contribute to the gap in CVD mortality rates between the two regions.

## Additional files

Additional file 1:
**Characteristics of included studies.**
Additional file 2:
**Summary of results of included studies.**
Additional file 3:
**Search terms used for MEDLINE search.**

## Abbreviations

CV: Cardiovascular disease
CEE: Central and Eastern Europe
DAFNE: Data Food Networking
EFSA: European Food Safety Authority
EHIS: European Health Interview Survey
EPIC: European Prospective Investigation into Cancer and Nutrition study
FAO: Food and Agriculture Organization
FBS: Food balance sheet
FFQ: Food frequency questionnaire
FSU: Former Soviet Union
HAPIEE: Health Alcohol and Psychosocial Factors in Eastern Europe study
HBS: Household budgetary Survey
STROBE: Strengthening the Reporting of Observational Studies in Epidemiology
WE: Western Europe
