# Supplementary material for: Does the consumption of fruits and vegetables differ between Eastern and Western European populations? Systematic review of cross-national studies
Source: Arch Public Health. 2015 Jun 15;73(1):29. doi: 10.1186/s13690-015-0078-8 (PMC4466869; doi:10.1186/s13690-015-0078-8)
Supplement: Additional file 1: — Characteristics of included studies. [file 13690_2015_78_MOESM1_ESM.pdf]

Table 2. Characteristics of included studies

1st author, year of publication	Name of study	Examined food or antioxidant	Dietary assessment	Participants` country of origin	Year of data collection	Month of data collection	Sample size	Response rate (%)	Females (%)	Age range or mean (years)	Sampling method	Basis of sample	Quality score* (max:18)
1. DIETARY SURVEYS													
Kromhout 1989(22)	Seven Countries Study	Fruits, vegetables	7d record	CEE: Yugoslavia	1960-64	Jan-May, Sep	150	nd	0	40-59	random	farm/factory workers, academics	9
				WE: Finland, Italy, Greece Netherlands	1959-65	Feb-Sep	286	nd	0	40-59	random	village inhabitants, railroad workers	
Winkler 1992(31)		Fruits, vegetables	3d record	CEE: GDR	1987	Oct-Dec	132	73	0	45-64	random	urban inhabitants	11
				WE: FDR	1984-85	Oct-May	424	70	0	45-64	cluster	urban inhabitants	
Schroll 1996(23)	SENECA	Fruits, vegetables	Diet history	CEE: Poland	1993	Jan-Jun	120	51†	61	74-79	random	urban inhabitants	13
				WE: Belgium, Denmark, France, Italy, Netherlands, Portugal, Spain, UK, Switzerland	1993	Jan-Jun	1237	51†	51	74-79	random	urban inhabitants	
Karamanos 2002(24)		Fruits, vegetables	Diet history	CEE: Bulgaria	nd	nd	288	nd	50	35-60	random	urban inhabitants	14
				WE: Italy, Greece	nd	nd	1058	nd	54	35-60	random	urban and rural inhabitants	
Serra-Majem 2003(25)	WHO-CINDI	Fruits, vegetables	24hr recall	CEE: Poland	1991-94	nd	4440	nd	50	20-65	random	factory workers	14
				WE: Spain	1992	nd	2757	69	nd	6-75	random	general population	
Petkeviciene 2009(26)	NORBAGREEN	Fruits, vegetables	FFQ	CEE: Lithuania	2002	Apr	99	68	57	19-75	random	general population	15
				WE: Finland	2002	Jan-May	125	91	nd	25-64	random	general population	
Lixandru 2010(32)		Fruits, vegetables	FFQ	CEE: Romania	2005	Apr-Nov	40	nd	30	63	convenience	diabetic patients	12
				WE: Belgium	2005	Apr-Nov	30	nd	20	62	convenience	diabetic patients	
Paalanen 2011(33)		Fruits, vegetables	FFQ	CEE: Russia	1992-07	Mar-May	2672	45-92	57	25-64	random	general population	16
				WE: Finland	1992-02	Mar-May	4365	67-81	53	25-64	random	general population	
Crispim 2011(34)	EFCOVAL	Fruits, vegetables	24hr recall	CEE: Czech Republic	2007-08	Oct-Apr	118	nd	51	45-65	convenience	healthy individuals	16
				WE: Belgium, France, Norway Netherlands,	2007-08	Apr-Jul, Oct-Apr	482	nd	50	45-65	convenience	healthy individuals	
El Ansari 2012(35)	CNSHS	Fruits, vegetables	FFQ	CEE: Bulgaria, Poland	2005	nd	1143	95	70	21	convenience	university students	14
				WE: Denmark, Germany	2005	nd	1236	85-92	53	21	convenience	university students	

To be continued

1st author, year of publication	Name of study	Examined food or antioxidant	Dietary assessment	Participants` country of origin	Year of data collection	Month of data collection	Sample size	Response rate (%)	Females (%)	Age range or mean (years)	Sampling method	Basis of sample	Quality score* (max:18)
2. HEALTH BEHAVIOUR SURVEYS													
Wardle 1997[36]	EHBS	Fruits	na	CEE: Poland, Hungary, GDR	1989-92	nd	2293	90-100	51	22	convenience	university students	13
				WE: Austria, Belgium, FDR, UK Denmark, Finland, Spain, France, Greece, Iceland, Ireland, Italy, Sweden, Netherlands, Norway, Portugal, Switzerland	1989-92	nd	14192	90-100	56	21	convenience	university students	
Prattala 2007[37]	Finbalt Health Monitor project	Fruits	na	CEE: Estonia, Latvia, Lithuania	1998-02	Apr-May	15740	62-80	57	20-64	random	general population	16
				WE: Finland	1998-02	Apr-May	9354	65-70	53	20-64	random	general population	
Prattala 2009[38]	EUROTHIENE	Vegetables	na	CEE: Estonia, Latvia, Lithuania	2000-04	nd	14219	60-73	58	20-64	random	general population	15
				WE: Finland, Denmark, Spain, Germany, France, Italy	1998-04	nd	86924	61-87	51	20-64	random	general population	
Hall 2009[39]	WHS	Fruits, vegetables	na	CEE: Bosnia and Herzegovina, Croatia, Czech Republic, Estonia, Georgia, Hungary, Kazakhstan, Latvia, Russia, Slovakia, Slovenia, Ukraine	2002-03	nd	22475	69-100	53	18-99	random	general population	15
				WE: Spain	2002-03	nd	5448	86	60	18-99	random	general population	
European Commission 2013[40]	EHIS	Fruits, vegetables	na	CEE: Bulgaria, Czech Republic, Estonia, Latvia, Hungary, Poland, Romania, Slovakia, Slovenia	2006-09	nd	85921	56-89	53	15-99	random	general population	na
				WE: Belgium, Greece, Spain, France	2006-09	nd	62700	60-96	55	15-99	random	general population	
Burisch 2014[41]	ECCO-EpiCom	Fruits, Vegetables	na	CEE: Croatia, Czech Rep, Estonia, Hungary, Lithuania, Moldova, Romania, Russia	2010	Jan-Dec	249	76†	42	15+	Convenience	IBD patients (at diagnosis)	16
				WE: Cyprus, Denmark, Finland, Greece, Iceland, Ireland, Israel, Italy, Portugal, Spain, Sweden, UK	2010	Jan-Dec	933	76†	46	15+	Convenience	IBD patients (at diagnosis)	

To be continued

1st author, year of publication	Name of study	Examined food or antioxidant	Dietary assessment	Participants` country of origin	Year of data collection	Month of data collection	Sample size	Response rate (%)	Females (%)	Age range or mean (years)	Sampling method	Basis of sample	Quality score* (max:18)
3. ANTIOXIDANT STUDIES													
Kardinaal 1993[29]	EURAMIC	Beta-carotene in adipose tissue	na	CEE: Russia	1991-92	nd	200	79-97	0	51	convenience	hospital patients, healthy controls	16
				WE: Finland, Germany, Netherlands, Norway, UK, Spain, Switzerland	1991-92	nd	1180	50-98	0	54	convenience	hospital patients, healthy controls	
Kristenson 1997[42]	LiVicordia	Beta-carotene in plasma	na	CEE: Lithuania	1993-94	Oct-Jun	100	83	0	50	random	urban inhabitants	14
				WE: Sweden	1993-94	Oct-Jun	95	83	0	50	random	urban inhabitants	
Bobak 1998[27]		Beta-carotene in plasma	na	CEE: Czech Republic	1992	Sep-Nov	136	70	49	40-59	random	urban inhabitants	14
				WE: UK	1991-93	nd	358	73	31	40-59	random	civil servants	
Bobak 1999[43]		Beta-carotene in plasma	na	CEE: Czech Republic	1995	Apr-Jun	188	70	0	45-64	random	general population	17
				WE: Germany	1995	Apr-Jun	153	70	0	45-64	random	general population	
Miere 2007[44]		Vitamin C intake	24h recall	CEE: Romania	nd	nd	312	nd	87	21	convenience	university students	8
				WE: Spain	nd	nd	918	nd	58	22	convenience	university students	
Woodside 2013[45]	EUREYE	Vitamin C and Beta-carotene in plasma	na	CEE: Estonia	2000-03	nd	833	58.6	66	65+	random	general population	15
				WE: Norway, UK, France, Italy, Greece, Spain	2000-03	nd	3300	36-56	52	65+	random	general population	

WHO-CINDI, World Health Organization Countrywide Integrated Non-communicable Disease Intervention; NORBAGREE, Consumption of vegetables and fruits and other dietary health indicator foods in the Nordic and Baltic countries; EFCOVAL, European Food Consumption Validation; CNSHS, Cross National Student Health Survey; EHBS, European Health and Behaviour Survey; WHS, World Health Survey; EHIS, European Health Interview Survey; EURAMIC, European Community Multicentre Study on Antioxidants, Myocardial Infarction and Breast Cancer; LiVicordia, Linkoping-Vilnius Coronary Disease Risk Assessment Study; ECCO-EpiCom, European Crohn`s and Colitis Organization`s Epidemiological Committee study; FDR, Federal Republic of Germany; GDR, German Democratic Republic; CEE: Central and Eastern Europe (or Former Soviet Union); WE, Western Europe; FFQ, Food frequency questionnaire; na, not applicable; nd, no data available; IBD, Inflammatory bowel disease

*Based on evaluation using a modified STROBE checklist;

†Overall response rate
