# Supplementary material for: Does the consumption of fruits and vegetables differ between Eastern and Western European populations? Systematic review of cross-national studies
Source: Arch Public Health. 2015 Jun 15;73(1):29. doi: 10.1186/s13690-015-0078-8 (PMC4466869; doi:10.1186/s13690-015-0078-8)
Supplement: Additional file 2: — Summary of results of included studies. [file 13690_2015_78_MOESM2_ESM.pdf]

Table 3. Summary results of the included studies

1st author, year of publication	Unit of measurement	Sex	CEE countries			WE countries			Power	SUMMARY: CEE compared to WE‡
			Average intake, cc. or %	Range*	SD	Average intake, cc. or %	Range*	SD		
1. DIETARY SURVEYS										
FRUITS										
Kromhout 1989[22]§|	g/day intake	M	58.6	1.0-153.6	207.3†	132.1	21.3-310.9	178.3†	0.96	LOWER
Winkler 1992[31]	g/day intake	M	98.0		145.3	101.0		164.3	0.05	no difference
Schroll 1996[23]§	g/day intake	M	186.0		239.1†	234.0	120.0-532.5	230.2†	0.26	lower-ns
		F	162.0		210.2†	208.0	135.0-399.6	202.4†	0.43	lower-ns
Karamanos 2002[24]	g/day intake	M	293.0		239.1†	315.0	236.0-355.0	239.1†	0.16	no difference
		F	303.0		210.2†	325.7	234.0-377.0	210.2†	0.21	lower-ns
Serra-Majem 2003[25]§	g/day intake	M+F	137.0		224.7†	290.0		218.0†	1.00	LOWER
Petkeviciene 2009[26]	p/month intake	M+F	20.8		84.3†	29.4		84.3†	0.12	no difference
Lixandru 2010[32]	% eat daily	M	100.0		na	89.5		na	0.34	higher-ns
		F	100.0		na	100.0		na	na	no difference
Paalanen 2011[33]	% eat daily	M	14.0	2.0-31.0	na	52.3	43.0-61.0	na	1.00	LOWER
		F	26.0	4.0-50.0	na	73.3	66.0-82.0	na	1.00	LOWER
Crispim 2011[34]	g/day intake	M	207.0		176.7	197.0	163.0-228.0	175.1	0.07	no difference
		F	226.0		155.7	230.5	194.0-265.0	151.1	0.05	no difference
El Ansari 2012[35]	% eat daily	M	31.6	23.8-39.4	na	30.4	28.6-32.1	na	0.05	no difference
		F	46.8	39.5-54.1	na	51.6	47.8-55.4	na	0.42	lower-ns

To be continued

1st author, year of publication	Unit of measurement	Sex	CEE countries			WE countries			Power	SUMMARY: CEE compared to WE‡
			Average intake, cc. or %	Range*	SD	Average intake, cc. or %	Range*	SD		
VEGETABLES										
Kromhout 1989[22]§ |	g/day intake	M	240.0	159.0-276.0	198.2†	102.6	57.3-227	88.1†	1.00	HIGHER
Winkler 1992[31]	g/day intake	M	126.0		154.8	124.0		154.8	0.05	no difference
Schroll 1996[23]§	g/day intake	M	341.0		154.8†	288.0	82.4-461.0	128.1†	0.63	higher-ns
		F	297.0		143.9†	238.0	77.0-383.0	121.0†	0.92	HIGHER
Karamanos 2002[24]	g/day intake	M	243.0		154.8†	189.0	168.0-214.0	154.8†	0.96	HIGHER
		F	291.0		143.9†	197.3	178.0-222.0	143.9†	1.00	HIGHER
Serra-Majem 2003[25]§	g/day intake	M+F	288.0		149.4†	97.1		68.7†	1.00	HIGHER
Petkeviciene 2009[26]	p/month intake	M+F	29.9		56.0†	29.1		56.0†	0.05	no difference
Lixandru 2010[32]	g/day intake	M	287.0		189.4	269.9		108.1	0.07	no difference
		F	258.3		157.9	283.3		125.2	0.06	no difference
Paalanen 2011[33]	% eat daily	M	15.0	10.0-24.0	na	48.7	44.0-54.0	na	1.00	LOWER
		F	22.3	11.0-35.0	na	70.7	69.0-72.0	na	1.00	LOWER
Crispim 2011[34]	g/day intake	M	162.0		121.1	201.0	168.0-222.0	112.8	0.60	lower-ns
		F	157.0		99.1	202.3	166.0-254.0	108.5	0.87	LOWER
El Ansari 2012[35]	% eat daily	M	37.8	23.9-51.6	na	24.4	23.3-25.4	na	0.99	HIGHER
		F	44.9	28.0-61.8	na	42.0	37.5-46.4	na	0.18	no difference

To be continued

1st author, year of publication	Unit of measurement	Sex	CEE countries			WE countries			Power	SUMMARY: CEE compared to WE‡
			Average intake, cc. or %	Range*	SD	Average intake, cc. or %	Range*	SD		
2. HEALTH BEHAVIOUR SURVEYS										
FRUITS										
Wardle 1997[36]	% eat daily	M	40.0	36.0-45.0	na	42.9	23.0-78.0	na	0.43	lower-ns
		F	65.0	59.0-74.0	na	61.1	36.2-86.0	na	0.72	higher-ns
Prattala 2007[37]	% eat daily	M	11.0	10.0-12.0	na	18.0		na	1.00	LOWER
		F	20.3	17.0-25.0	na	36.0		na	1.00	LOWER
EHIS 2013[40]	% eat daily	M	52.8	39.4-66.8	na	60.6	57.9-66.0	na	1.00	LOWER
		F	67.0	49.2-82.3	na	69.1	62.3-74.5	na	1.00	LOWER
Burisch 2014[41]	% eat daily	M+F	43.4		na	54.3		na	0.87	LOWER
VEGETABLES										
Prattala 2009[38]	% eat daily	M	22.5	16.1-27.5	na	32.1	24.7-39.1	na	1.00	LOWER
		F	30.4	25.0-33.4	na	45.9	36.9-59.1	na	1.00	LOWER
EHIS 2013[40]	% eat daily	M	54.8	44.2-71.3	na	68.6	56.0-82.7	na	1.00	LOWER
		F	62.5	55.0-78.6	na	74.2	65.3-87.4	na	1.00	LOWER
Burisch 2014[41]	% eat daily	M+F	49.0		na	60.1		na	0.88	LOWER
FRUITS and VEGETABLES										
Hall 2009[39]	% eat >=5 p/day	M	18.1	8.0-44.5	na	22.0		na	0.98	LOWER
		F	23.5	9.4-49.7	na	24.9		na	0.38	lower-ns

To be continued

1st author, year of publication	Unit of measurement	Sex	CEE countries			WE countries			Power	SUMMARY: CEE compared to WE‡
			Average intake, cc. or %	Range*	SD	Average intake, cc. or %	Range*	SD		
3. ANTIOXIDANT STUDIES										
BETA CAROTENE										
Kardinaal 1993[29]	ug/g fatty acid	M	0.51	0.45-0.56	0.80	0.42	0.18-0.59	0.80	0.31	higher-ns
Kristenson 1997[42]	umol/l cc.	M	0.38		0.20	0.51		0.32	0.92	LOWER
Bobak 1998[27]	umol/l cc.	M	0.39		0.26†	0.77		0.26†	1.00	LOWER
		F	0.52		0.40†	0.97		0.40†	1.00	LOWER
Bobak 1999[43]	umol/l cc.**	M	0.11		0.08	0.20		0.21	1.00	LOWER
Woodside 2013[45]	umol/l cc	M	0.25		0.26	0.34	0.19-0.48	0.31	1.00	LOWER
		F	0.36		0.34	0.44	0.30-0.67	0.37	1.00	LOWER
VITAMIN C										
Miere 2007[44]	mg/day intake	M	80.3		54.8	106.2		83.4	0.77	lower-ns
		F	88.8		67.9	124.4		94.8	1.00	LOWER
Woodside 2013[45]	umol/l cc.	M	42.0		23.8	38.0	32.7-44.4	23.1	0.74	higher-ns
		F	54.5		27.7	48.5	43.5-52.4	23.4	1.00	HIGHER

M, Males; F, Females; p, portion; EHIS, European Health Interview Survey; na, not applicable; cc., concentration;

*Range of intake levels, percentages or concentrations if data was reported from more than one country or site;

†SD assumed from EPIC study;

‡ LOWER: Intake level, percentage or concentration significantly lower in CEE/FSU countries compared to data from WE, (power>0.80); HIGHER: Intake level, percentage or concentration significantly lower in CEE/FSU countries compared to data from WE, (power>0.80); lower-ns: Intake level, percentage or concentration lower in CEE/FSU but difference not significant (power<0.80 and >0.20); higher-ns: Intake level, percentage or concentration higher in CEE/FSU but difference not significant (power<0.80 and >0.20); no difference: power<0.20

§: North-South weighting was applied

I: Seasonal weighting was applied

** Calculated from reported data using molar mass=537g
