# Supplementary material for: Does the consumption of fruits and vegetables differ between Eastern and Western European populations? Systematic review of cross-national studies
Source: Arch Public Health. 2015 Jun 15;73(1):29. doi: 10.1186/s13690-015-0078-8 (PMC4466869; doi:10.1186/s13690-015-0078-8)
Supplement: Additional file 3: — Search terms used for MEDLINE search. [file 13690_2015_78_MOESM3_ESM.doc]

**Additional file 3** Search terms used for MEDLINE search

exp Europe, Eastern/ OR exp USSR/ OR exp Czechoslovakia/ OR exp Germany, East/ OR exp Yugoslavia/ OR exp Transcaucasia/ OR exp Asia, Central/ OR central europe*.mp. OR eastern europe*.mp. OR alban*.mp. OR armen*.mp. OR azerbajan*.mp. OR belarus*.mp. OR bosnia*.mp. OR hercegovina*.mp. OR bulgar*.mp. OR croat*.mp. OR czechslovak*.mp. OR czech*.mp. OR east german*.mp. OR eston*.mp. OR georgia*.mp. OR hungar*.mp. OR kazakh*.mp. OR kyrgiz*.mp. OR latvia*.mp. OR lithuan*.mp. OR montenegro*.mp. OR poland*.mp. OR polish*.mp. OR moldova*.mp. OR roman*.mp. OR russia*.mp. OR serb*.mp. OR slovak*.mp. OR sloven*.mp. OR tajik*.mp. OR macedon*.mp. OR turkmen*.mp. OR ukrain*.mp. OR soviet*.mp. OR uzbeg*.mp. OR ussr*.mp. OR yugoslav*.mp.

AND exp Nutritional physiological phenomena/ OR exp Vegetables/ OR exp Fruit/ OR exp Carotenoids/ OR exp Ascorbic acid/ OR vegetable*.mp. OR fruit*.mp. OR caroten*.mp. OR lycopen*.mp. OR ascorbic*.mp.

AND exp Epidemiologic Methods/ OR exp multicenter study/ OR exp comparative study/

limit to humans

mp=title, abstract, original title, name of substance word, subject heading word, keyword heading word, protocol supplementary concept, rare disease supplementary concept, unique identifier, text word

exp …/ = Explode MeSH term
